# Surveillance systems for neglected tropical diseases: global lessons from China’s evolving schistosomiasis reporting systems, 1949–2014

**DOI:** 10.1186/1742-7622-11-19

**Published:** 2014-11-25

**Authors:** Song Liang, Changhong Yang, Bo Zhong, Jiagang Guo, Huazhong Li, Elizabeth J Carlton, Matthew C Freeman, Justin V Remais

**Affiliations:** Department of Environmental and Global Health, College of Public Health and Health Professions, and Emerging Pathogens Institute, University of Florida, 1225 Center Drive, Gainesville, FL 32611 USA; Sichuan Center for Disease Control and Prevention, Institute of Public Health Information, 6 Middle School Road, Chengdu, Sichuan, 610041 China; Sichuan Center for Disease Control and Prevention, Institute of Parasitic Diseases, 6 Middle School Road, Chengdu, Sichuan, 610041 China; Department of Schistosomiasis, Institute of Parasitic Diseases. Chinese Center for Disease Control and Prevention, Shanghai, China; Department of Control of Neglected Tropical Diseases, World Health Organization, Geneva, Switzerland; Department of Emergence Response, Chinese Center for Disease Control and Prevention, Beijing, China; Department of Environmental and Occupational Health, Colorado School of Public Health, University of Colorado, Aurora, CO USA; Department of Environmental Health, Rollins School of Public Health, Emory University, 1518 Clifton Rd. NE, Atlanta, GA 30322 USA

**Keywords:** Neglected tropical diseases, Parasitic disease, Case ascertainment, Schistosomiasis, Surveillance, Sampling, China

## Abstract

Though it has been a focus of the country’s public health surveillance systems since the 1950s, schistosomiasis represents an ongoing public health challenge in China. Parallel, schistosomiasis-specific surveillance systems have been essential to China’s decades-long campaign to reduce the prevalence of the disease, and have contributed to the successful elimination in five of China’s twelve historically endemic provinces, and to the achievement of morbidity and transmission control in the other seven. More recently, an ambitious goal of achieving nation-wide transmission interruption by 2020 has been proposed. This paper details how schistosomiasis surveillance systems have been structured and restructured within China’s evolving public health system, and how parallel surveillance activities have provided an information system that has been integral to the characterization of, response to, and control of the disease. With the ongoing threat of re-emergence of schistosomiasis in areas previously considered to have achieved transmission control, a critical examination of China’s current surveillance capabilities is needed to direct future investments in health information systems and to enable improved coordination between systems in support of ongoing control. Lessons drawn from China’s experience are applied to the current global movement to reduce the burden of helminthiases, where surveillance capacity based on improved diagnostics is urgently needed.

## Introduction

Schistosomiasis poses a major public health threat in many tropical and sub-tropical countries—the disease is endemic to 78 countries worldwide and, in 2011, at least 243 million people were infected and needed treatment [1]. In China, despite substantial progress achieved reducing the burden of schistosomiasis over the past two decades, the disease remains an ongoing public health challenge [2, 3]. Based on the most recent data, nearly 250,000 people are estimated to be infected in 171 counties in China [4]. Since the 1950s, endemic areas have been identified in 454 counties in 12 provinces [3, 4]. As of 2012, transmission had been eliminated in 5 of the 12 endemic provinces, and morbidity and transmission control had been attained in the other 7 (see official control categories and their criteria in Table 1; [5, 6]). The country has set an ambitious goal of achieving nation-wide transmission interruption by 2020, however several obstacles lie in the way, including re-emergence in previously controlled areas [7], as well as challenges achieving lasting interruption of transmission in remaining endemic foci [3, 4].

**Table 1:** **Criteria for assigning county-level schistosomiasis transmission status**

Transmission status	Criteria
Infection control	Human infection prevalence <5%
	Bovine infection prevalence <5%
	<10 acute cases over 2 week period in a village
Transmission control	Human infection prevalence <1%
	Bovine infection prevalence <1%
	No acute cases
	No infected snails (*O. hupensis*) for two consecutive years
Transmission interruption	No human cases for five consecutive years
	No bovine cases for five consecutive years
	No snails (*O. hupensis*) for two consecutive years
Elimination	No new infection in humans or bovines for five years after reaching transmission interruption

Note: All criteria are evaluated at the administrative village-level.

China’s multiple, evolving surveillance systems for schistosomiasis have been integral to the country’s decades-long campaign to reduce the prevalence of the disease. Chinese surveillance for schistosomiasis began in the middle of the 20th century after the founding of the People’s Republic, with identification of endemic areas through both hospital-based reporting and limited epidemiological surveys. Currently, there are four parallel surveillance systems specific to schistosomiasis (Table 2)—*routine* surveillance, *national* surveys, *sentinel* surveillance and the national infectious disease reporting system (*NIDRS*). These systems serve distinct, but complementary purposes. The *routine* surveillance system (termed ‘routine surveys’ in some literature), was initiated in the 1950s. The other three schistosomiasis surveillance systems created in the late 1980s were strengthened considerably in 1992 when the Chinese government received a long-term World Bank loan aimed at improving schistosomiasis morbidity control. The loan resulted in significant investment in schistosomiasis research [8] and disease control efforts, as well as financial support for the *national* and *routine* surveillance systems [8, 9].

**Table 2:** **Summary of key characteristics of current schistosomiasis surveillance systems in China**

	NIDRS	Sentinel	Routine	National
**Active/passive**	Passive	Active	Active	Active
**Year initiated**	1950s (1989 for schistosomiasis)	1990	1950s	1989
**Reporting unit**	Individuals in hospitals	Sentinel village	Village	Village
**Coverage**	All hospitals	Nine sentinel villages	All villages in endemic counties	1% of villages in endemic provinces
**Time frame**	Real-time, within 24 hours of patient diagnosis	Yearly	All villages sampled over ~3 years, reporting occurs at completion of each village’s survey	Periodic every 6-9 years: 1989, 1995, 2004
**Purpose**	Aid understanding of disease patterns; provide evidence for policy-making	Longitudinally and objectively monitor how the schistosome-endemic situation changes over time	Evaluate control measures	Clarify the endemic status of schistosomiasis as established by the previous national survey
**Information collected**	Individual cases (demographics, patient residence, diagnosis, treatment and hospital)	Snail habitat, human infection prevalence and intensity, bovine infection prevalence	Snail habitat, human infection prevalence	Human infection prevalence and intensity, bovine infection prevalence, snail habitat
**Diagnostics**	Clinical and laboratory	IHA screen then Kato-Katz and miracidium hatch	IHA screen then Kato-Katz	ELISA screen then Kato-Katz
**Major changes**	2004: Replaced paper-based monthly or yearly reporting with internet-based real-time reporting system	2011: Added Miracidium Hatch Test to diagnostic procedure	2011: Replaced yearly reporting with internet-based parasitic disease reporting that occurs after completion of each village survey	2004: Inclusion criteria expanded to include areas with prevalence >0.5% from previous criterion of >1%
**Strengths**	Inexpensive, Algorithms can be created to automatically detect outbreaks of emerging or reemerging disease	Provides longitudinal measures of disease prevalence and intensity	Provides greatest coverage since it samples all endemic villages in the province	Provides a nationwide estimate of schistosomiasis prevalence
**Limitations**	Underreporting of chronic cases; potential underreporting of acute cases due to political pressure; potential information bias associated with variable clinical and diagnostic capacities of reporting sites; non-response bias associated with reporter fatigue	Sampling occurs at limited sites (20 in 1989, 80 in 2005); longitudinal follow-up over decades can yield non-response bias resulting from participation and reporter fatigue; potential selection bias associated with the choice of villages to sample longitudinally	Variable clinical and diagnostic capabilities can lead to information bias; potential selection bias associated with choice of survey sites, since those in charge of surveillance are also in charge of control efforts; potential reporting bias as funding can be tied to disease control success; potential non-response bias resulting from participation fatigue and temporary rural-to-urban migration	Occurs rarely and survey methods change, making it difficult to assess temporal patterns; only includes 1% of endemic villages in each province

IHA: indirect hemagglutination assay; Village: administrative village, with typical population of ~1000 people.

Schistosomiasis, like many neglected tropical diseases and other human helminthiases in particular, presents several surveillance challenges. First, the disease is typically concentrated in low-income, rural areas where health infrastructure is limited. Second, the clinical presentation of the disease is rarely acute, and like many chronic diseases, long-term infections can evade clinical detection and eventually lead to severe health sequelae later in life. Thus reliance on passive hospital or clinic-based reporting can grossly underestimate the number of infections. China’s long history conducting schistosomiasis surveillance through multiple systems presents a unique opportunity to examine how their complementary structures, designs and sampling approaches—and their evolution over time—have provided essential epidemiological information to support the control of a neglected tropical disease.

The goal of this paper is to detail how China's schistosomiasis surveillance systems have been structured and restructured as part of China’s evolving public health system, yielding a combined information system that has been integral to the country's progress in reducing the burden of the disease. We discuss the diagnostic approaches underlying these systems, as well as additional tools necessary for cost-effective schistosomiasis surveillance in endemic settings that are approaching transmission control and elimination but face the threat of re-emergence. Finally, we discuss ways in which China’s experience with schistosomiasis surveillance can offer insights to other countries as they develop and strengthen their own infectious disease surveillance systems.

### Diagnostic methods for detecting schistosomiasis infections in humans and animals

China’s schistosomiasis surveillance programs target three key hosts: humans, bovines and snails. Assays to detect schistosomiasis infections in human hosts are used to provide a direct measure of disease burden in the population, whereas surveys of bovines and snails (spatial distribution and infections) provide a means to evaluate the transmission potential of a given setting, and the particular role played by non-human reservoirs.

### Detection of human infections

Detection of schistosomiasis infections in humans in China is typically accomplished by immunologic or coprologic assays. Immunologic assays commonly used in China include *Schistosoma japonicum*-specific immunoglobulin G standardized enzyme-linked immunosorbent assay (ELISA) and the indirect hemagglutination assay (IHA). Diagnostic immunological test kits have historically been subsidized by the national government, which has contributed to their widespread use. From a practical standpoint, immunologic methods are desirable as they require only a blood sample from a finger or ear-stick. Immunoassays generally have high sensitivity, but low specificity, due in part to the inability of the assay to distinguish between past and current infections [10]. The immunological tests are generally used as a first-step screening method in large population surveys: those who test positive by immunoassay are then tested with a coprologic exam.

China relies on the Kato-Katz technique as the primary coprologic schistosomiasis diagnostic, following guidance of the World Health Organization [11]. The method involves the microscopic examination of a stool sample smeared across three slides using a light microscope. Slides are examined by trained laboratory technicians who count *Schistosoma* eggs in standardized 41.7 mg smears and calculate eggs per gram (EPG) of the original sample, providing a widely accepted proxy measure of infection intensity [12]. The assay lacks sensitivity in low infection-intensity regions [13, 14].

Another common coprologic assay is the miracidium hatch test, which detects the presence of viable schistosome eggs in stool by inducing miracidia to hatch in an aqueous solution. Typically performed in county-level anti-schistosomiasis stations, 30 g stool samples are strained through a 200 μm nylon mesh to concentrate schistosome eggs and remove small particles. The enmeshed material is then suspended in an aqueous solution, and placed in a lit, temperature-controlled environment where the sample is examined at two, five and eight hours for the presence of miracidia using low magnification [14].

### Detection of bovine infections

Detection of *S. japonicum* in the bovine population serves as a complement to human surveillance, as bovines are a key non-human reservoir of *S. japonicum* in China. Thus detection of infections in bovines offers a means of monitoring the role of non-human hosts in introducing or sustaining transmission, particularly in agricultural settings where bovines are abundant [14]. To test bovines for infection, a variant of the miracidium hatch test is used with modified observation times. Because the high water content of bovine stool can promote rapid hatching, the suspended sample is typically examined at shorter time intervals (one, two, and four hours) after preparation relative to the human exam [15].

### Quantification of intermediate host densities and infections

Assessments of the density and infection status of the intermediate snail host *Oncomelania hupensis* have long played an important role in China’s surveillance for schistosomiasis. Snail density surveys consist of collecting and counting snails from historical or newly identified snail habitat. Specimens are drawn from a consistent *kuang*-sized (0.11 m^2^) sampling frame at random or equal interval points along streams, irrigation canals, fields, and lakes in endemic and some formerly endemic areas [7]. The infection status of sampled specimens is determined by microscopic examination for the presence of cercariae. This method is limited in that it is only capable of detecting patent infections, and even in regions where human infection prevalence is high, the prevalence of *S. japonicum* infections in snails is very low (<<1%; [16]), and thus screening of a large number of snail hosts is needed. In areas where human infection prevalence is low, infected snails are rare and a poor indicator of the presence of human infections [14].

### Future diagnostic needs

As schistosomiasis infection prevalence declines in China, more sensitive diagnostic methods are needed [14]. Recent diagnostic innovations include methods that detect *S. japonicum* DNA in human and bovine stool or serum, as well as in snails [17–19], but these assays have not yet been widely adopted. Such approaches offer potentially greater sensitivity than existing methods, particularly in areas where infection intensities are low, which is a key technological need for schistosomiasis elimination [20]. Issues of cost, standardization, laboratory infrastructure and technical training remain to be addressed for this new class of molecular diagnostic techniques.

### History of institutional support for schistosomiasis surveillance in China

In 1956, a massive nationwide schistosomiasis control campaign was launched, which bore the slogan "Schistosomiasis has to be eliminated" and was subsequently memorialized by Chairman Mao Zedong’s poem "Farewell to the God of Plague" in which he mourned the advance of the disease and championed its subsequent retreat [21, 22]. Medical school graduates were required to spend time in rural areas, both to provide care directly as village doctors, as well as to train local residents to serve as paramedics, or so-called *barefoot doctors*[23, 24]. At the same time, China established administrative and professional health organizations at varying levels of government to carry out both surveillance and control tasks aimed at eliminating specific diseases. These included an *Office of Endemic Disease* (OED) in each province, which designed and managed surveillance and control programs, as well as units targeting specific infections. For schistosomiasis, county-level anti-schistosomiasis stations were established under the supervision of OEDs and were responsible for carrying out surveys on humans and the snail intermediate host, control activities and health education. These stations established the necessary infrastructure and recording procedures for documenting infection and treatment statuses, disease progression of patients, and distribution and abundance of the intermediate host of the pathogen, and hence played a central role in the first standardized schistosomiasis surveillance activities [7].

In the late 1970s, reforms to China’s health systems led to the dismantling of the cooperative health system in place of a market-based system, and by 1985, barefoot doctors either became village doctors who worked on a fee-for-service basis, or they shifted to other professions [25]. This substantial change in government policy led to a dramatic decline in primary health care coverage in rural areas. The restructuring of the public health system also involved the establishment of Epidemic Prevention Stations (EPS) at provincial, prefecture and county administrative levels, with a vertical structure for disease reporting and for the provision of technical guidance [26]. Provincial, prefecture, and county EPS were financed by the health bureau at the same administrative level, resulting in little administrative control of higher-level EPS over lower-level institutions (although technical guidance did flow from higher- to lower-level units). Prefecture and county EPS primarily reported to their local health bureau instead of provincial EPS or the Ministry of Health. This structure would change dramatically with the most recent major overhaul of China’s heath systems at the turn of the 21^st^ century [26, 27].

**Figure 1:**
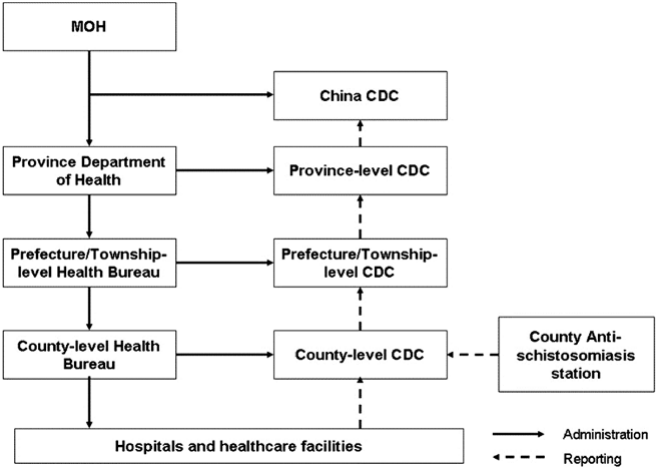
**Structure of China’s Public Health System after 2002** [44] **.**

In 2002, the National Center for Disease Control and Prevention and Control (China CDC) was established from the former Chinese Academy of Preventive Medicine [27]. With the founding of China CDC, nearly all institutions responsible for disease control were integrated into a single system that linked national, provincial, prefecture and county levels. All levels of EPS were converted to CDCs and linked with other CDCs in a vertical structure that persists to this day (Figure 1; [27]).

### China’s evolving schistosomiasis surveillance systems

Surveillance was spotty and inconsistent in the years following the founding of People’s Republic of China, and not until 1989 did robust schistosomiasis surveillance programs emerge [28]. A massive, national survey in 1989 confirmed endemic counties and assessed schistosomiasis prevalence status in 12 provinces based on human infections and an assessment of intermediate host habitat [29]. Starting in 1992, additional schistosomiasis surveillance systems came online, including annual, locally administered surveys in endemic villages, as well as intensive national surveys to estimate the prevalence of schistosomiasis across provinces and in several high priority areas [30]. In 2003, the SARS epidemic exposed key weaknesses in China’s ability to detect and manage infectious disease epidemics [31]. In response, the Chinese government invested heavily in an enhanced National Infectious Disease Reporting system (NIDRS), which launched in 2004. The overhauled NIDRS was fully internet-based, and the number of reportable diseases was increased to reflect the country’s need for key surveillance data to support infectious disease prevention and control programs. At the same time, China’s entire public health system was being streamlined as described above, leading to the formation of China CDC [32].

Coincident with this massive transformation of China’s public health institutions, a large World Bank loan that supported schistosomiasis control programs was ending, and its completion in 2001 resulted in a significant loss of funding for schistosomiasis surveillance. Surveillance and control efforts decreased, contributing to the re-emergence of schistosomiasis in several locations where it had previously been controlled [7, 33]. In response, the Chinese Ministry of Health included schistosomiasis on a list of four infectious diseases targeted with high priority for surveillance and control, along with tuberculosis, HIV/AIDS and Hepatitis B. This was a major policy change, establishing schistosomiasis control as a national priority and elevating the position of the disease on the list of nationally notifiable diseases (i.e., from class C to class B; Table 3). Improvements in schistosomiasis surveillance followed, as increased commitment and funding flowed from the central government.

**Table 3:** **Infectious diseases covered by mandatory** ***NIDRS*** **reporting** [31]

Rank	# of diseases	Reporting time	Diseases
Class A	2	2 hours	Plague, Cholera
Class B	26	24 hours^§^	SARS^§^, HIV/AIDS, Viral hepatitis (A, B, C, E, other), Poliomyelitis^§^, Human avian influenza^§^, Measles, Epidemic hemorrhagic fever, Rabies, Epidemic Japanese encephalitis B, Dengue fever, Anthrax^§^, Tuberculosis, Dysentery (viral or amebic), Epidemic cerebrospinal meningitis, Typhoid and Paratyphoid, Pertussis, Diphtheria, Tetanus neonatorum, Scarlet fever, Brucellosis, Gonorrhea, Syphilis, Leptospirosis, Schistosomiasis, Malaria, H1N1 swine flu
Class C	11	24 hours	Influenza, Mumps, Rubella, Acute hemorrhagic conjunctivitis, Leprosy, Typhus, Leishmaniasis, Echinococcosis, Filariasis, Infectious Diarrhea other than cholera, dysentery or typhoid and paratyphoid, Foot-and-mouth disease

^§^Cases of SARS, poliomyelitis, pulmonary anthrax and human infection with highly pathogenic avian influenza must be reported within 2 hours.

In the following sections, we describe the purpose, sampling approach, and diagnostics used in each of the four schistosomiasis surveillance systems—*routine* surveys*, national* surveys, *sentinel* surveys and *NIDRS*—and we describe how each surveillance system was impacted by changes in China’s health systems following the SARS epidemic in 2004.

### Routine surveys

*Routine* schistosomiasis surveys involve periodic infection screening in every endemic village in the seven endemic provinces. Staff at county-level anti-schistosomiasis stations have historically conducted these screenings in collaboration with local village governments and hospitals [7]. *Routine* surveys generate data used by the Ministry of Health to evaluate control measures, and provide information used by provincial-level schistosomiasis control steering committees to determine which counties have satisfied the official criteria for transmission control (Table 1). The primary outcomes and other characteristics of *routine* surveys are summarized in Table 2.

### Changes since 2004

With the substantial increase in central government support for schistosomiasis control since 2004 [34], local anti-schistosomiasis stations began receiving funding directly from the central government to carry out and improve *routine* surveys, and to implement interventions based on survey findings. Because anti-schistosomiasis stations are responsible for both surveillance and control, and the funding they receive from the national government is in part correlated to control success, reporting bias may be present in *routine* surveillance data. The national and provincial health bureaus set annual goals (e.g., prevalence reduction targets) for anti-schistosomiasis stations, which were folded into annual control performance reviews for the stations. To improve reliability of these surveys, a real-time, internet-based reporting system was established in 2011 to file, store and manage *routine* survey data [35], and quality control checks are routinely performed on these electronic records to detect and reduce reporting biases.

### Sampling methodology

*Routine* surveys in a given endemic county are typically conducted over the course of two, three or more years depending on the level of endemicity in—and the control status of—the county. For counties that have achieved transmission control (Table 1), all villages are surveyed over a three-year period (i.e., one-third of villages in the county are surveyed each year). In counties that have achieved transmission interruption, all villages are surveyed over four- or five-year periods, and in counties that have yet to achieve transmission control, all villages are surveyed over two years. Villages are randomly selected and grouped into two (for counties not yet achieving transmission control), three (for counties achieving transmission control), or four or five (for counties achieving transmission interruption) subgroups. Each year, one subgroup of villages is surveyed, and this is repeated annually until all subgroups are surveyed. At that point, villages are randomly grouped into new subgroups, and the multi-year survey process is repeated. In villages that are part of the *sentinel* schistosomiasis surveillance system (see below), *routine* surveys are not conducted and the necessary information is shared from the *sentinel* system.

In a given village, *routine* surveillance involves intermediate host surveys in the spring followed by surveys of the human population in the fall, at the end of the transmission season. The target human sample is >90% of residents between the ages of 6 and 65 years old [9]. Sampling of cattle and other alternative mammalian hosts are also included in *routine* survey efforts, but these are the responsibility of the local animal husbandry department and the degree of coordination between the veterinary and public health efforts is highly variable [7]. All residents are requested to present themselves at a designated location for screening on the day of the survey. Villager participation in these surveys has been inconsistent. Residents may not feel ill and therefore may see no need to be tested for schistosomiasis. In endemic areas, surveys have been completed every year or every other year for >50 years, leading to participation fatigue. To overcome this challenge, health workers have conducted health education campaigns that emphasize the impacts of undetected, chronic infections, and the importance of routine schistosomiasis examinations. When residents are missed by a *routine* survey screening, public health workers will seek them out in their homes and request their participation.

Sampling and screening protocols for *routine* surveys are standardized by the Ministry of Health to improve consistency between counties and provinces, though variations have been noted. In Sichuan Province, for example, different diagnostics have been used between counties, some carrying out serologic exams only, some serologic followed by stool exams and some stool exams only. Thus, there are limitations when directly comparing *routine* survey data between counties.

### Relationship to routine surveillance systems for other parasites and diseases

In China, *routine* surveys are unique to schistosomiasis, providing spatial and temporal data on infection patterns that are generally unavailable for other parasitic diseases. The surveys are very resource-intensive, and thus they have not been adopted, for instance, for soil-transmitted helminths (STHs). A sentinel STH system exists and resembles *routine* schistosomiasis surveys with less exhaustive sampling. The standardized training of county-level staff that carry out *routine* survey screenings—and the infrastructure developed to support them—have provided ongoing quantitative data on the distribution of cases at fine spatial scales [36]. These data are unique to schistosomiasis, and have provided essential information to schistosomiasis reduction programs throughout China.

### National surveys

In 1989, China carried out the first *national* schistosomiasis survey with the purpose of estimating the prevalence of the disease in each endemic province. The resulting information served as an important baseline dataset for the World Bank loan for schistosomiasis control (see Introduction), and the 1989 data were subsequently compared with the 1995 *national* survey in an economic valuation of the loan-financed programs [37]. *National* surveys are large, periodic cross-sectional studies that generate estimates of prevalence among humans and domestic animals across endemic settings. They are carried out every 6–9 years in China, and their primary outcomes and other characteristics are summarized in Table 2.

### Changes since 2004

The most recent *national* schistosomiasis survey in 2004 included considerable technical improvements over previous surveys, such as an additional sampling stratum targeting low intensity infection areas; use of ultrasonography to screen a small number of subjects tested for schistosome-induced fibrosis; use of a subset of surveyed villages to assess the sensitivity of the Kato-Katz test as performed by Chinese technicians [28]; and sampling to estimate snail density and snail infection prevalence in one county within each province [37]. Importantly, the 2004 *national* survey included all formerly endemic areas that had achieved infection control *and* transmission control (see Table 1), whereas previous surveys focused on formerly endemic areas that had achieved infection control only. These and other changes to the *national* survey sampling design in 2004 provided important information as China oriented its control programs towards national elimination, but also generated survey results that were not directly comparable to the 1989 or 1995 surveys [28].

### Sampling methodology

**Figure 2:**
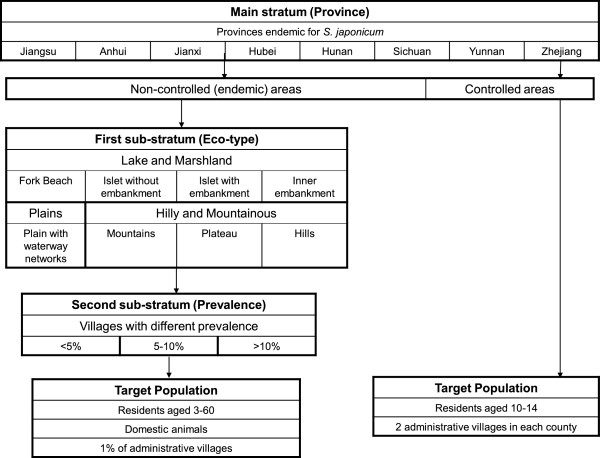
**Sampling design for the 1989 and 1995** ***national*** **schistosomiasis surveys** [36]**.** A stratified cluster random sampling design with three strata was used. The first sampling stratum includes the seven schistosomiasis-endemic provinces (Jiangsu, Anhui, Jiangxi, Hubei, Hunan, Sichuan, and Yunnan) and one controlled province (Zhejiang). For endemic provinces, environmental and ecosystem characteristics were used to define the first sub-stratum (eco-type), and the level of prevalence defined the second sub-stratum (prevalence). A target survey population was drawn from each of the second sub-strata, with the characteristics defined in the figure. For areas that have achieved control (Zhejiang province in the 1989 and 1995 *national* surveys), the target survey population was drawn from two administrative villages from each historically endemic county.

*National* surveys are mandated by the Chinese Ministry of Health, but are designed, managed and overseen by the OEDs in the seven endemic provinces [28]. The sampling unit is endemic administrative villages, as defined by a combination of data from the preceding *national* survey and preceding years of *routine* survey data. The sampling design is based on a stratified cluster random sampling across three strata: (1) province, (2) environment/ecotype, and (3) estimated local prevalence based on the most recent *routine* survey data (Figure 2). All residents 6 to 65 years old are eligible to participate in *national* surveys, and the ELISA method is used for initial screening, followed by Kato-Katz exams to confirm and quantify infection for all ELISA positives [28].

Because *national* surveys use standardized sampling methodologies, the results are comparable between provinces. What is more, the Ministry of Health uses the *national* surveys’ representative population sampling strategy to estimate prevalence across each endemic province while decreasing the required size of the sample. However, the sampled population, chosen in part based on historical prevalence, may not be representative [28].

### Relationship to national surveys for other parasitic diseases

The Chinese government has historically carried out national surveys for other human parasitic diseases [38]. Between 1988 and 1992, and again between 2001 and 2004, national surveys tested for 56 different parasites, including protozoa and helminths [28, 39], using a random sampling scheme and a set of diagnostics similar to the *national* schistosomiasis surveys [40]. However, sampling for these national surveys was not informed by routine surveillance data. Thus, in the absence of recent prevalence data, socioeconomic factors—including variables such as sanitation access, income and literacy—were used to stratify the population for sampling.

### *Ad hoc* provincial surveys following *national* survey protocols

As an example of how new surveillance capacity can follow on existing efforts, some provinces carry out *provincial* schistosomiasis sampling surveys borrowing sampling methodology from the 1989 and 1995 *national* surveys, but with the purpose of including a larger population within the province to boost sensitivity. Provincial OEDs design provincial survey sampling protocols, including human, bovine and intermediate host surveys, and surveys are carried out by anti-schistosomiasis stations in each county with the support of the EPS. Endemic villages from each county are sampled randomly within three categories based on infection prevalence and infection intensity determined from prior *routine* surveys, as follows. One third of endemic villages are randomly selected from each of three categories of infection prevalence based on results from *routine* surveys: less than five percent prevalence; between 5 and 15 percent; and >15 percent. In settings with low variability in infection prevalence between villages, sampling is based on infection intensities, with one third of villages sampled each from light, medium and heavy infection categories, again based on the results of recent *routine* surveys. In Sichuan’s provincial survey in 2001, for instance, 1,188 villages were sampled, involving >1,810,000 people ranging from age 5 to 65. The survey took six months to complete, from April to October, and all cattle were screened in each sampled village. For comparison, for the *national* surveys in Sichuan, >52,000 people from 39 villages, and >51,000 people from 40 villages, were surveyed respectively in 1989 and 1995. The more intensive sampling regimen of the provincial survey provided more detailed spatial coverage than the *national* survey.

### National sentinel surveillance

The *sentinel* schistosomiasis surveillance system was initiated in 1989 with 20 sites in the seven endemic provinces, selected to represent different eco-epidemiological zones of transmission [9]. The purpose of the *sentinel* system is to monitor longitudinal prevalence and infection intensity. *Sentinel* surveys consist of human and bovine infection screening—along with snail surveys—with the purpose of capturing temporal changes in prevalence and intensity of infection [9]. For example, in Sichuan Province there are four counties enrolled in *sentinel* surveillance—Xichang, Dangling, Guanghan and Pujiang—where snail sampling takes place two times a year, in late spring and in fall, and human and animal surveys are conducted in the fall [9]. Within each county, one village is selected each year where at least 90% of village residents over the age of six are sampled.

### Changes since 2004

The *sentinel* system expanded in 2005 to include 80 sites across China, reflecting the Chinese government’s increased commitment and funding to schistosomiasis surveillance and control. In Sichuan Province, the number of sentinel sites increased from four to nine, and of these, six are in hilly regions, one is from a plateau region, and two are from mountainous areas. Hilly areas are represented with the most sentinel sites because infections in Sichuan are most common in these regions. The nine Sichuan sites have remained constant since 2005, and there is a commitment to continue longitudinal tracking of schistosomiasis infections in these sites regardless of their transmission trajectories in order to take maximum advantage of a consistent longitudinal dataset.

Diagnostic protocols for the *sentinel* system have changed to accommodate the changing transmission profiles of sentinel sites, particularly challenges in detecting low-intensity infections. As of 2011, for instance, sentinel sites in Sichuan Province test all those classified as positive using a serologic assay with both Kato-Katz and a miracidium hatch test. These multiple rounds of diagnoses, and a history of intensive surveillance generally in these historically endemic regions, have led to community-wide surveillance fatigue in some sentinel sites. Costs of implementing the ongoing *sentinel* surveillance are likely to increase as it becomes more challenging to achieve high levels of community participation, necessitating more intensive follow-up with residents and more sensitive diagnostic methods.

### Sampling methodology

The number of sentinel sites sustained by the system is based on the level of funding provided by the central government, and provinces with higher prevalence and infection intensity are allotted more resources (and thus sites). To select sites, national and provincial level OEDs consider two factors: variation in infection intensity (sites are selected from communities with heavy, medium and light infections based on infection intensity measurements from prior *routine* surveillance); and eco-epidemiological zone (e.g., plateau, lake, hilly or mountainous regions). *Sentinel* system screening has been conducted by Kato-Katz since 1989, and the IHA test was added in 2000, with patients found positive by IHA confirmed with the Kato-Katz exam. If acute or advanced cases are found, epidemiological investigations are conducted to collect more detailed information about each case.

The overall design of the *sentinel* system was developed and approved by a national steering committee, yielding a system that is consistent among all surveillance sites, and making the data comparable between sites in all seven endemic provinces. It is sometimes necessary to move sites when implementing an intensive longitudinal surveillance system. For instance, after >10 years at one site in Sichuan, the population exhibited fatigue from yearly surveys and a new site was substituted to increase community participation. In one instance, as infection intensity and prevalence decreased dramatically across Sichuan Province, a very low prevalence sentinel site was replaced with a high prevalence site. Substituting sites interrupts the *sentinel* surveillance time-series, and data at new sites provide few insights into temporal trends until the series is allowed to accumulate over a number of years.

### National infectious disease reporting system

The National infectious disease reporting system (*NIDRS*) was established in the 1950s [31], but it was not until 1989, with the passing of China’s Law on Preventing and Treating Infectious Diseases, that the reporting of selected infectious diseases was mandated by law [32]. The *NIDRS* has historically involved a vertical reporting structure where hospitals reported the aggregated number of cases monthly by post to the county health department, who in turn report aggregate cases to the prefecture, then to the province, and finally to the central government. The information is used by Chinese public health officials to understand national patterns of infectious disease transmission, and to develop appropriate prevention and control programs [41].

### Changes since 2004

Following the SARS outbreak, China’s Law on Preventing and Treating Infectious Diseases was revised, and the *NIDRS* was transformed into an internet-based, real-time system [31]. The new system involves the routing of individual cases (rather than strictly aggregated data) up the hierarchy of public health institutions. The system provides for timely reporting of schistosomiasis cases to public health officials, allowing for rapid identification of, and response to, suspected re-emergence of the disease. The reporting of individual cases also enhances the spatial and temporal resolution of disease reporting, allowing for assessment of, for example, seasonal trends and persistent hot-spots of transmission [31].

### Sampling methodology

Cases of schistosomiasis diagnosed in clinics or hospitals are reported directly to the *NIDRS*. Reporting includes both acute and chronic cases, however acute schistosomiasis contributes a disproportionate number of reported schistosomiasis cases. Acute schistosomiasis is a severe allergic reaction to the migrating schistosomule in the blood stream following infection and typically occurs only in naïve populations such as children or military personnel deployed to a new region. The rapid onset of severe symptoms typical of acute schistosomiasis is more likely to prompt health-seeking behavior, therefore resulting in more frequent capture by *NIDRS*[42]. Because the symptoms of chronic *S. japonicum* infection are non-specific and often sub-clinical, chronic infections are poorly captured by clinic-based surveillance. Thus *NIDRS* schistosomiasis surveillance is considered key for identifying areas of disease emergence (or re-emergence), but not for assessing infection prevalence. What is more, diagnosis of a reported case is coded in the *NIDRS* as using either a clinical or a laboratory diagnostic, without further differentiation such as between stool and serologic exams [43]. The primary outcomes and other characteristics of the *NIDRS* system are listed in Table 2.

Schistosomiasis diagnostic and clinical capabilities, and therefore reporting capacity, differ considerably across China, and the transition to the internet-based *NIDRS* revealed several limitations of surveillance capacity in China [31]. The availability of computing resources and internet coverage required to connect directly to *NIDRS* varied within and between provinces, limiting the generation and transmission of electronic case reports [44]. Hospitals that lack sufficient information technology resources were required to send paper forms to the county CDC for entry into the online system, introducing a reporting delay. A national initiative was launched to equip such hospitals with computer workstations and network coverage, resulting in the delivery of >4,000 computers to hospitals and clinics in Sichuan Province in 2005, for example [45]. Other challenges were revealed relating to disparities in clinical resources. For instance, a disproportionate number of the cases of schistosomiasis present in *NIDRS* have been reported by county-level hospitals, in part because diagnostic capabilities are poorer at township-level hospitals, and diagnostic capabilities for parasitic diseases are sometimes entirely absent at private clinics and village healthcare centers [46].

### Relationship to reporting systems for other diseases

At present, the *NIDRS* covers 39 infectious diseases in three classes based on their public health importance (Table 3; [47]). The Class A list includes two diseases, cholera and plague, that are required to be reported within two hours of diagnosis. Pulmonary anthrax, SARS, poliomyelitis, and human infection with highly pathogenic avian influenza are in Class B, and are also required to be reported within two hours. Other Class B and Class C diseases must be reported within 24 hours of diagnosis. Class C diseases include newly identified diseases or diseases with emerging public health importance, including foot and mouth disease. Schistosomiasis was listed as a Class B nationally reportable infectious disease in 2004 [47], in part to rapidly capture reports of *acute* cases that may signal an outbreak of infection [43].

### Future directions in surveillance

Ideally, surveillance systems provide data that support evidence-based policy decisions and contribute to effective and efficient disease control [48, 49]. Additionally, effective surveillance systems must respond to advances in technology and changes in programmatic goals. While multiple, complementary systems can provide rich spatial and temporal information on the distribution of cases, resource constraints necessitate surveillance systems that minimize redundancies, particularly in low- and middle-income countries.

Key factors in the evolution of China’s schistosomiasis surveillance over the past six decades include the World Bank loan (and the rise in cases following its termination in 2001); changes in the epidemiology of schistosomiasis; changes in available technologies for diagnosis and treatment [50]; and major public health events such as the 2003 SARS epidemic. The SARS epidemic in particular revealed weaknesses in China’s public health system with respect to the detection of, and prompt response to, disease outbreaks [31]. Following the epidemic, the country’s infectious disease surveillance infrastructure underwent a massive overhaul, which included construction of new infectious disease facilities and laboratories that were crucial to improving the quality of surveillance data, as well as facilitation of improved data sharing and of more efficient coordination between public health agencies. The overhaul also included a complete redesign of the real-time infectious disease reporting system [51], as well as the Ministry of Health's listing of schistosomiasis among diseases such as HIV/AIDS, tuberculosis and hepatitis B as a high priority for control [25]. This classification raised the profile of the disease among public health officials, increased the commitment of health care workers and government agencies to schistosomiasis elimination, and stimulated heightened surveillance and control efforts [43, 52].

Throughout these major transitions, China has made remarkable progress towards controlling schistosomiasis. The country’s evolving, parallel surveillance systems have each played a distinct role in characterizing the dynamic state of schistosomiasis transmission across diverse regions of the country. Thus far, the systems and the information they provide have been used independently; numerous reports, based on *routine* surveys, *sentinel* surveys and *national* surveys have been published and used to develop control strategies and monitor progress towards achieving elimination. More recently, there has been an effort to expand the use of these data beyond endemic disease control. *Routine* surveillance data, for instance, has been used to provide evidence of re-emerging schistosomiasis in Sichuan Province in areas that had previously achieved local control [7]. Surveillance data from multiple systems can be fruitfully combined, as evidenced by integration of *national* and *NIDRS* surveillance data from all provinces to estimate the incidence, prevalence and disease burden of schistosomiasis, as well as malaria, hookworm and other water, sanitation, and hygiene-associated infections [53]. The analysis revealed large regional disparities in the burden of these infections, particularly among children, who experience the greatest risk of water, sanitation and hygiene-attributable disease in China. In a separate study, combined *national* and *NIDRS* data were used to estimate the future burden of these infections under climate change [54]. While this work demonstrates the value of China’s parallel surveillance systems, understanding of how data from these systems can be effectively combined, to characterize and inform the many dimensions of disease transmission and control, remains limited.

The overlapping nature of these systems, both spatially and temporally, provides a unique opportunity to explore *how* a country can maximize the value of multiple, distinctive information sources to inform public health campaigns and investments. This in an area where future research is needed both in China and elsewhere. Furthermore, as many provinces move towards schistosomiasis elimination and case detection becomes ever more challenging and expensive, it will be even more important to determine how the data, sampling approaches, and detection methods these systems provide can be used to optimize the performance of surveillance efforts. Successful schistosomiasis elimination will require the ability to efficiently and accurately identify the few remaining infected individuals and non-human reservoirs among large uninfected populations. This will be complicated by decreased diagnostic sensitivity owing to reduced infection intensity, testing fatigue among residents as a result of long-standing repeated examinations, and ever-increasing health care costs associated with diagnosis. To be successful, China’s surveillance system must be prepared to modify sampling methods, reporting approaches and diagnostics in response to new scientific and technological advances and changing schistosomiasis infection patterns. Recent attempts to establish diagnostic standards through the systematic evaluation of an array of immunological assays [55] and new mobile reporting methods, discussed below, are a promising step in this direction. Further, as China's national disease burden continues its shift from infectious diseases to chronic, non-communicable diseases (NCDs), additional strain will be placed on surveillance personnel and institutions currently devoted to infectious disease, and opportunities for integrating the surveillance and care of NCDs and infectious diseases will be needed [56, 57]. Care must be taken to ensure that the resources are adequate and to avoid surveillance fatigue among healthcare workers and those in sentinel villages.

For China, research and innovations needed to succeed in continuing control and elimination of schistosomiasis include the development of diagnostic tools with higher sensitivity for the detection of human infections, and new ways of assessing human susceptibility to infection. In addition, effective ways to deploy recently developed tools for detecting the infectious stage of schistosomes (cercariae) in the natural environment [30, 58, 59] will be needed. Likewise, mobile disease reporting technologies (e.g. use of mobile phones or positioning systems with wireless capability for spatially explicit reporting of cases or vectors) show considerable promise and have been recently applied for surveillance of other neglected tropical diseases [60]. These technologies have been used in field surveys of schistosomiasis endemic areas, to file reports of acute cases and infected snails into a centralized surveillance database [9], and to coordinate responses to disease outbreaks after the 2008 earthquake in Sichuan province [16, 61]. Yet effective integration of mobile technologies into large-scale surveillance procedures is still relatively new, and will need further evaluation. At the same time, new approaches will be needed to estimate the cost-effectiveness of the combination of *existing* surveillance systems in achieving specific surveillance endpoints, such as a characterization of spatial and temporal variability in cases. While considerable effort has been devoted to evaluating surveillance systems from a functional standpoint [62–66] —assessing timeliness [67], record accuracy [68, 69] and enhancing operational efficiency [70–72] —few analytical tools exist for quantitatively assessing how changes to design translate into improved (or inferior) estimates of the underlying rates of disease taking into account system sensitivity, coverage, bias and other factors. Development of such tools in China would provide a useful precedent as schistosomiasis surveillance is increasingly brought online in other countries.

### Global lessons

Globally, there is a major movement underway to reduce the burden of helminthiases. The priorities for the global control and elimination of schistosomiasis, along with STHs, were articulated by the WHO in 2013 [73]. The surveillance needs of these global efforts are immense. Nearly 240 million people, most of whom live in sub-Saharan Africa, are estimated to require preventive chemotherapy for schistosomiasis alone as of 2009, yet coverage is reaching only 8% of those in need [74, 75]. While many factors contribute to this deficit, the lack of surveillance capabilities in nearly all schistosomiasis-endemic countries is a major obstacle to efficiently expanding coverage [74]. Accurate, timely, country-level data are generally unavailable, and targeting drug delivery for schistosomiasis control in these settings is predominantly based on risk maps generated using ad-hoc epidemiological data (e.g., research data, historical drug delivery data, etc.), rather than surveillance [76]. China’s history with surveillance of schistosomiasis can serve as a guide for countries, in sub-Saharan Africa and elsewhere, that require low-cost, simple surveillance and diagnostic technologies.

Given the current efforts to roll-out preventative chemotherapy, there will be a need to identify areas where disease burden is declining and mass-treatment efforts can be transitioned to targeted treatment and monitoring, as well as areas where disease burden is not declining, where more broad control efforts are needed. Throughout Africa, where over 90% of schistosomiasis cases occur [77], the capacity to conduct population-based surveys is limited [78], and current control recommendations do not include surveillance activities [74]. There is some movement towards developing the infrastructure for parallel active surveillance systems—sentinel systems to track progress of control strategies coupled with regular provincial surveys—in highly endemic areas. China’s *sentinel* systems offer a model for deploying targeted surveillance resources and monitoring control progress over time. Similar to China’s provincial surveys, which borrow from standardized *national* survey protocols, province-level schistosomiasis mapping of all endemic districts has recently begun in a few countries (e.g. Ethiopia), and is rapidly scaling up for other neglected tropical diseases such as trachoma, with the benefit of funds provided by the UK Department for International Development and The Bill & Melinda Gates Foundation [79]. These efforts are moving incrementally towards the electronic collection and reporting of epidemiological data, and may benefit from lessons learned during China’s 2011 adoption of a simple, internet-based system to file, store and manage *routine* survey data. Crucially, this system provides a model for electronic data collection even in the absence of a sophisticated *NIDRS* capacity. As many low-resource countries aspire for control and even elimination of schistosomiasis, understanding the synergy of parallel systems will prove cost-effective both by reducing unnecessary redundancy in data collection and in maximizing programming dollars. The systems described in China have distinct utility, but how these systems may be translated to other areas of schistosomiasis endemicity is not yet known and must be explored. Looking forward, targeted surveillance capacity is needed to understand and respond to the transmission consequences of rapid environmental, social, and technological changes, such as the construction of dams and changes in agricultural practices, that can result in an upsurge in schistosomiasis cases [54, 77, 80–82]. China’s *sentinel* system offers a model for deploying targeted surveillance capacity in regions undergoing such changes [83].

Optimal surveillance of schistosomiasis and other neglected tropical diseases must evolve with changing programmatic needs and technical advances. Currently, real-time electronic surveillance is limited by lack of broadband internet infrastructure in many rural schistosomiasis-endemic areas. However, mobile disease reporting technologies offer new opportunities in areas where traditional means of communication are limited [6], and may become increasingly relevant as attention turns to elimination. In the context of elimination, new diagnostic tools are urgently needed that offer rapid, sensitive, low-cost alternatives to current methods. China is currently dealing with this issue [14], and as other settings move towards achieving elimination, the traditional diagnostics that are currently relied upon by many surveillance systems will need to be replaced [84]. Recently developed methods offer improved opportunities for point-of-care surveillance (e.g., [85]), and future investments will be needed to ensure advances like these are pursued and, ultimately, incorporated into surveillance practice.
